# Sleepwalking and prolonged partial sleep paralysis in a case of obstructive sleep apnea

**DOI:** 10.5935/1984-0063.20200053

**Published:** 2021

**Authors:** Sudheer Tale, Akash Kumar, Lokesh Kumar Saini, Soibam Pahel Meitei, Ravi Gupta

**Affiliations:** 1 All India Institute of Medical Sciences Rishikesh, Pulmonary Medicine - Rishikesh - Uttarakhand - India.; 2 All India Institute of Medical Sciences Rishikesh, Psychiatry - Rishikesh - Uttarakhand - India.

**Keywords:** Obstructive Sleep Apnea, Sleepwalking, Sleep Paralysis, Somnambulism, Parasomnias

## Abstract

Observation of episodes of sleepwalking and prolonged partial sleep paralysis in the same patient is a rare condition. We present a case of 42 years gentleman who presented with recurrent episodes of sleepwalking and prolonged incomplete sleep paralysis. He was on tablet divalproate 1000mg/day and tablet olanzapine 5mg/day in view of a psychotic episode that occurred 4 years ago. Polysomnography suggested presence of moderate obstructive sleep apnea (OSA) with intrusion of alpha waves in sleep. Considering only one psychotic episode with no other risk factors, these medications were gradually tapered and discontinued. Symptoms improved after tablet clonazepam 0.5mg at bedtime even while he was not compliant to continuous positive airway pressure (CPAP).

## INTRODUCTION

Parasomnias are specific group of sleep disorders that includes sleep talking, sleepwalking, and night terrors. These are associated with repeated arousals, usually occurring in non-rapid eye movement (NREM) sleep^1^. Similarly, rapid eye movement (REM)-related parasomnias include REM-sleep behavior disorder, recurrent isolated sleep paralysis, and nightmare disorder^1^. NREM parasomnias are common and obstructive sleep apnea is a risk factor for development of NREM parasomnias, especially if arousal-threshold is low^1^.

Sleep-paralysis is usually short lasting and profound, however, at times it may be incomplete making diagnosis difficult^2^. Sleep paralysis is caused by intrusion of REM related atonia in wakefulness and is usually short lasting^1^. We are presenting a rare case where patient presented with sleepwalking along with partial and prolonged sleep paralysis with obstructive sleep apnea. Treatment of clonazepam even while he was not compliant to continuous positive airway pressure (CPAP) therapy brought relief in parasomnia episodes.

## CASE REPORT

A 42-year-old male presented with complaints of sudden awakenings from sleep since past 16 months. During awakenings, he was engaged in complex behaviors; however, characteristics were different for episodes. During first incident, he woke after 3 hours of sleep onset followed by a fall on the floor leading to forehead injury. Though he appeared oriented but was irritable for 1-2 hours and then fell asleep. Next morning, he woke up normally but could not recall the event. Similar episode was repeated after about a year, but was briefer, lasting for 10-15 min.

In another episode, which occurred after 3 hours of fall asleep, he went to kitchen and micturated on the wall. After voiding, he came back and went back to sleep. In some other incidences he found himself sleeping at different places in his house in the morning. However, he could not recall any of these episodes. During other few episodes, he woke up and went to other room, where he switched on the lights and laid on the bed. When he was woken up by wife during the episode, he appeared confused for some time but did not have memory for the episode later. He reported some tiredness in the morning following the nights when these incidents would happen.

For the past 6 months, he reported waking up with bad dreams as if he had fallen down and trapped. This was followed by awakening and recognition that “it was a dream”. At that time, he would try to get out of bed but he would not be able to stand up and would feel extreme weakness in bilateral lower limbs. He had to be supported by his wife to get up to the bed. This would last around 10 to 20 min and he would remain aware of surroundings during these episodes. These episodes occurred after 3-4 hours of falling asleep and recurred 1-2 times a month.

There is also history of snoring during sleep for the past 3 to 4 years. He gives history of waking up multiple times at night, feeling thirsty and breathlessness. He would also go to the toilet at least 3 to 4 times a night as compared to once or twice during daytime.

He was using alcohol in dependent pattern for 9 years. However, he had become sober since past 5 years with occasional alcohol use, which was unrelated to any of the above incidents. There appears to be a psychotic episode 4 years back when he was prescribed tablet divalproex 1000mg/day and olanzapine 5mg/day. He was maintaining well on these medications and did not have any symptoms while presented to us. Considering just one episode of psychosis and no other risk factors, these medications were gradually tapered 2 weeks before and discontinued one day before the diagnostic polysomnography. He met a road traffic accident and suffered major head injury 2 years back. However, he regained consciousness after 4 days without any major neurological deficit.

There was no history of shift work, irregular sleep schedule, diabetes, hypertension, pulmonary tuberculosis, and asthma. He did not report any dizziness, vertigo, lightheadedness, blurred vision, weakness in other parts of body associated with episodes of weakness in lower limbs. Family history was negative for snoring, NREM or REM parasomnias, hypersomnia, restless legs syndrome. There was no childhood history of any kind of sleep disorder.

On general craniofacial examination dental overjet and macroglossia were noticed along with high arched hard palate and Mallampatti score was grade IV. Body mass index was 25.7kg/m2 and there was no pedal edema. Other systemic examination was unremarkable. To differentiate between sleep related seizures and parasomnia, frontal lobe epilepsy and parasomnia (FLEP) scale was applied that showed score of minus 4 suggesting very unlikely to have epilepsy^3^. Magnetic resonance imaging (MRI) brain revealed encephalomalacia and gliosis involving left superior gyrus, left basilateral region and left temporal lobe with microbleeds in frontal gyrus.

**Figure 1:**
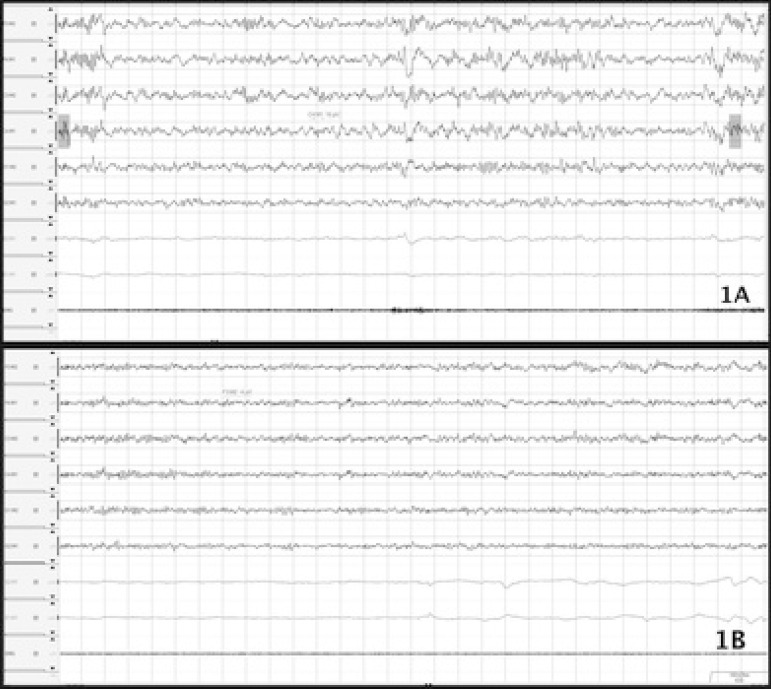
Epochs from the non-REM sleep and REM sleep (30 sec). A. Showing Alpha intrusion during N2 sleep; B. Muscle atonia during REM sleep.

Based on the information available, diagnosis of “sleepwalking with incomplete sleep paralysis with obstructive sleep apnea (OSA)” was made. Attended video-synchronized in-lab polysomnography was done. Important findings of sleep study are shown in Table 1. During diagnostic study, intrusion of alpha waves was seen during non-rapid eye movement 2 (N2) sleep and chin atonia was observed during REM sleep (Figure 1). He did not report experiencing any of these episodes during sleep study. Following night, he could not tolerate mask and hence, positive airway pressure (PAP) titration could not be done. He was discharged on tablet clonazepam 0.25mg at night. He did not report any episode since past 6 months.

**Table 1:** Main findings of the diagnostic sleep study.

Sleep variables			
Total sleep time	425 min		
Sleep onset latency	26.2 min		
REM latency (from sleep onset)	40.5 min		
Sleep efficiency	78.2 %		
**Sleep stages**			
N1	15.3 %		
N2	29.5 %		
N3	16.6 %		
REM	38.6 %		
Wake index (per hour)	3.4		
**Indices (per hour)**	**REM sleep**	**NREM sleep**	**Total sleep**
Arousal indices			
Arousal index (Total)	31.1	29.5	30.1
Spontaneous arousal index	18.7	15.9	17.0
Respiratory + Desaturation+ Snore arousal index	10.3	11.8	9.6
Leg movement arousal index	2.2	1.9	2.0
**Respiratory indices**			
Apnea hypopnea index	34.8	14.7	22.4
Desaturation index	------	-----	20.7
**Limb movement indices**			
Limb movement (total) index	25.2	7.4	22.0
Periodic limb movement index	6.6	1.6	9.0

## DISCUSSION

Two factors were interesting in this case-first differentiation between sleep related hyper motor epilepsy and NREM/REM parasomnia. Second, diagnostic clarification of episodes of weakness associated with the dreaming.

Briefer and multiple episodes usually favor sleep related seizures while opposite of it favors parasomnia^3^. Paroxysmal events during sleep in this case were of longer duration and were occurring infrequently. Moreover, FLEP scale has been found to have high specificity and positive predictive value to differentiate parasomnia from sleep related hyper motor epilepsy in two studies^3^^,^^4^. It has been argued that FLEP may mistake REM sleep behavior disorder with epilepsy; however, it was ruled out by maintenance of atonia during REM sleep (Figure 1B).

Moreover, intrusion of alpha waves during sleep (Figure 1A), presence of OSA (Table 1) and good response to clonazepam favored the diagnosis of NREM parasomnia^1^^,^^4^.

Pathophysiologically, NREM parasomnia appear owing to local arousal in cortical areas and recruitment of central pattern generators^1^. Patients with sleepwalking are often involved in complex motor activity and do not have any memory of the event after waking up^1^. Any condition that disrupts the continuity of sleep may induce NREM parasomnia, e.g., sleep-deprivation, stress, periodic limb movement disorder and obstructive sleep apnea to name a few^1^. Successful treatment of OSA with CPAP has been found to improve sleepwalking in patients having sleepwalking along with OSA^5^. In present case, patient was not compliant to CPAP. Hence, he was given tab clonazepam that is known to improve the maintenance of sleep and improve NREM parasomnia is a sizable number of patients^6^. Hence, it may be deduced that clonazepam increased the arousal threshold even in presence of OSA, thereby leading to resolution of symptoms.

Another interesting point in present case was that the patient developed NREM parasomnia after the age of 40 years, whereas the usual age of onset for the sleep walking or night terror is in childhood. This can be explained by the findings of Schenck et al.^7^, in 100 adult patients with sleep related injuries. They had found that almost one third of the patients had onset of night terror or sleep walking after the age of 16 years, while the maximum age of onset was 58 years^7^. This indicates that it is not uncommon to have a later age of onset for sleepwalking or night terror.

A significant challenge was to differentiate it from REM sleep behavior disorder (RBD) and NREM parasomnia^1^. RBD is characterized by violent dreams and enactment, which was not seen in this case, though this possibility cannot be ruled out considering onset of episode after 4 hours of sleep. Patients with RBD, sleep-terrors as well as sleep-paralysis report that they have woken up from sleep. However, weakness in the skeletal musculature after waking-up has not been reported either in RBD or sleep-terrors^1^. Patients with isolated sleep paralysis report waking up from sleep with inability to move body for a brief period owing to continuation of REM atonia during wakefulness, however, in this case it was incomplete and prolonged. Short REM latency in the present case (Table 1) could have resulted from chronic sleep deprivation as this patient had high arousal index^1^. Evidence of chronic sleep deprivation, waking up from the dream, unable to move for a significant period, presence of OSA and observed of REM atonia during polysomnography (Figure 1) were the factors that favored diagnosis of isolated sleep paralysis (ISP) over REM sleep behavior disorder^1^^,^^2^^,^^4^. ISP is usually profound and involves whole of the body. However, incomplete ISP is also known, although only one report could be found^2^. Awakening from a bad dream, having short lasting extreme weakness in bilateral lower limbs, awareness of surroundings during the episodes favor diagnosis of incomplete ISP^2^.

Antidepressants can ameliorate symptoms in cases of isolated sleep paralysis by blocking REM sleep^2^^,^^8^. However, we decided to try clonazepam as patient had history of alcohol use disorder and diagnosed OSA, both the conditions that disrupt continuity of sleep and pave way for sleep-walking as well as ISP^8^. Measures to improve sleep continuity are recommended to treat cases of ISP^8^. Clonazepam maintenance the continuity of sleep and improves parasomnias, especially NREM sleep parasomnia^9^. However, this has never been tried in ISP and present case emphasizes that clonazepam can be a good therapy for cases of ISP where sleep disruption is thought to induce it.

In conclusion, we are presenting a case where maintenance of sleep continuity improved sleepwalking and incomplete ISP, even when OSA remained untreated.
